# Paternity analysis, pollen flow, and spatial genetic structure of a natural population of *Euterpe precatoria* in the Brazilian Amazon

**DOI:** 10.1002/ece3.4582

**Published:** 2018-10-31

**Authors:** Santiago Linorio Ferreyra Ramos, Gabriel Dequigiovanni, Alexandre Magno Sebbenn, Maria Teresa Gomes Lopes, Jeferson Luis Vasconcelos de Macêdo, Elizabeth Ann Veasey, Alessandro Alves‐Pereira, Perla Pimentel da Silva, José Nivaldo Garcia, Paulo Yoshio Kageyama

**Affiliations:** ^1^ Instituto de Ciências Exatas e Tecnologia Universidade Federal do Amazonas Itacoatiara AM Brazil; ^2^ Departamento de Genética Escola Superior de Agricultura “Luiz de Queiroz”/Universidade de São Paulo (ESALQ/USP) Piracicaba SP Brazil; ^3^ Seção de Melhoramento e Conservação Genética Florestal Instituto Florestal de São Paulo São Paulo SP Brazil; ^4^ Faculdade de Ciências Agrárias Universidade Federal do Amazonas Manaus AM Brazil; ^5^ Embrapa Amazônia Ocidental Manaus AM Brazil; ^6^ Instituto de Pesquisa em Bioenergia Universidade Estadual Paulista Rio Claro SP Brazil; ^7^ Departamento de Ciências Florestais Escola Superior de Agricultura “Luiz de Queiroz”/Universidade de São Paulo (ESALQ/USP) Piracicaba SP Brazil

**Keywords:** açaí do Amazonas, coancestry coefficient, gene flow, paternity analysis, pollen and seed dispersal patterns, spatial genetic structure

## Abstract

*Euterpe precatoria*, known as açaí do Amazonas, is a regionally important palm of the Amazon rainforest for the fruit production through extractive agriculture. Little information is available with regard to genetic diversity, gene flow, and spatial genetic structure (SGS) of açaí populations, which are essential for the use, management, and conservation of genetic resources of the species. This research aimed to assess the genetic diversity, inbreeding level, SGS, and gene flow in four ontogenetic stages of a natural *E. precatoria* population in the Brazilian Amazon, based on 18 microsatellite loci. The study was carried out in a natural population dispersed in an area of about 10 ha. Leaf tissues of 248 plants were mapped and sampled and classified into four ontogenetic stages: reproductive (59), immature (70), young (60), and seedling (59). Genetic diversity indices were high for all ontogenetic stages. The fixation index (F) for all ontogenetic stages was not significantly different from zero, indicating the absence of inbreeding. A significant SGS was found for all ontogenetic stages (68–110 m), indicating seed dispersal over short distances. Paternity analysis detected pollen immigration of 39.1%, a selfing rate of 4.2%, and a mean pollen dispersal distance within the population of 531 m. The results indicate substantial allele input in the population via pollen immigration, contributing to the maintenance of the genetic diversity of the population. However, within a population, the renewal with new progenies selected from seed plants spaced at least 110 m apart is important to avoid collecting seeds from related plants.

## 1 INTRODUCTION

The protection of the Amazon forest ecosystem allows the conservation of tropical biodiversity and the sustainable management of non‐timber forest products (NTFPs), especially in poor regions, an alternative of social and economic development for local populations and indigenous communities. However, ecological impacts of NTFP exploitation may take longer to be perceived and require alternative approaches to be measured (Novello et al., 2018).

In the Amazon region, two NTFP species, *Euterpe precatoria* (Arecaceae) or “açaí do Amazonas” and *Euterpe oleracea*, are important palm trees in economic and social terms (Kahn, 1991) for the production of fruits of açaí by extractive farmers. Both species are processed and consumed the same way, whereas the main difference between these species is morphological: *E. precatoria* is single‐stemmed and *E. oleracea* multistemmed (Kahn, 1991; Kang et al., 2012). Both species have contributed to the fact that Brazilian Amazon is currently the largest açaí supplier on the national and international market. Between 2000 and 2010, the mean annual production of marketed açaí fruit was 117,063.59 tons and the State of Pará was the largest producing state (89.5% of the total production). From 2011 to 2016, the mean açaí fruit yield increased by 77.5%, that is, a production of 207,852.67 tons (Instituto Brasileiro de Geografia e Estatística [IBGE], 2018). The production increase was mainly due to the participation of the states of Pará and Amazonas; in average, the state of Pará accounted for 56.05% and the state of Amazonas for 33.91% of total production (IBGE, 2018). Geographically, in the state of Pará, açaí fruit is produced by *E. oleracea* and in the state of Amazonas by *E. precatoria* (Lorenzi, Noblick, Kahn, & Ferreira, 2010). After this increase in production by extractivist agriculture between 2011 and 2016, especially in the state of Amazonas, *E. precatoria* had in average an increase of 6,946.22% in these 6 years. Is this extractivist agriculture affecting the process of recruitment of new developing plants? and What production from these extractivist harvests is not yet being perceived for not presenting any ecological impacts?

Clearly, this species plays an essential role for the farmer and riverine populations, who depend on extractive exploitation. In the long term, local populations’ exploitation created a production chain, which is currently being expanded, including different industries. More farmers could easily have the opportunity to become part of this production chain, since *E. precatoria* is found all along the outskirts of the western Amazon and central Brazil, as well as along the Amazon border in Peru, Colombia (Kahn, 1991) and Bolivia (Bussmann & Zambrana, 2012; Food and Agriculture Organization of the United Nations [FAO], 1987). In the Amazon, *E. precatoria* occurs along rivers, in periodically flooded forests with alluvial soils (Henderson, 1995), due to the seasonal variation in water levels over the year (a water phase, during which the areas are flooded, and a terrestrial phase, when the areas are not flooded). This habitat contributes to the formation of different vegetation physiognomies, compositions, and structures, associated with physical and chemical characteristics of the soil, topography, and terrain slope (Marinho et al., 2013). The species is also adapted to nutrient‐poor and well‐drained latosols and ultisols (FAO, 1987), which can be exploited in the reforestation of disturbed or degraded areas. It requires annual rainfall levels between 1,900 and 4,000 mm, and a mean annual temperature of 26°C. In the forest (sub‐canopy), it can grow in environments with favorable or unfavorable soil, often forming small groups with 10–20 trees (FAO, 1987), for example, 50–250 plants/ha (Kahn, 1988), or it can be less abundant, with only 2–3 plants/ha (FAO, 1987). Thus, the distribution of *E. precatoria* can be considered wide and the density level of its populations classified as common (Kageyama et al., 2004).

The species is important too, particularly in rural communities in the Amazon region, where the trees are continuously used as building material, fabric, fuel, foods, medicine, and ornamental plants (Sosnowska & Balslev, 2009). The roots are used to prepare drugs to treat malaria, hepatitis, and other diseases (Bussmann & Zambrana, 2012). *Euterpe precatoria* is a highly promising species and should be valued accordingly, with investing efforts to initiate research on its domestication, particularly with regard to management and conservation techniques (in situ, ex situ*,* or *on farm*) in the short term, because this high demand can affect market supply and prices, aside from stimulating the implementation of monoculture areas of the species with little genetic variability.

In this context, monitoring of new developing *E. precatoria* plants in the forest, by paternity analysis, pollen flow, and spatial genetic structure (SGS) can be an effective way to assess the underlying impacts of harvest. This is a major concern for this species. This study will allow important information to toast the process of domestication, management, and conservation of *E. precatoria,* based on the assessment of the genetic diversity, inbreeding level, SGS, and gene flow. In general, pollen and seed dispersal patterns greatly influence the genetic structure and effective size of plant populations (Dow & Ashley, 1998), while in tropical forests, a good understanding of the ecosystem as a whole is also essential, underlying the establishment of reliable hypotheses to collect useful data of each species (Kageyama, Gandara, & Vencovsky, 2001). This information is important for genetic conservation, breeding, and possible plans for environmental recovery, because the mating patterns determine the relationship between and within families and, consequently, the effective size of each family (Murawski & Hamrick, 1991). The mating system and the pollen flow rate or an intensive dispersal within continuous populations can increase genetic diversity, the effective number of pollen donors and effective size within families, because the patterns and distance of pollen flow are important for the genetic diversity of populations (Hamrick, 2004).

This study with *E. precatoria* is the first to deal with the effective number of pollen donors, and to estimate the effective population size and establish an idea of the number of trees needed to collect seeds, which is key information for the conservation as well as cultivation of the species. The applied method was based on a categorical parentage analysis (Meagher, 1986), associated with highly polymorphic microsatellite markers (Ashley, 2010), which allowed the genealogical reconstruction of the relationship between plants within and between families (Manoel et al., 2012). The genetic diversity, inbreeding level, SGS, and gene flow were assessed in four ontogenetic stages of a natural *E. precatoria* population in the Brazilian Amazon, leading to the following questions: (a) Are the levels of genetic diversity, inbreeding, and SGS of the studied ontogenetic stages different? (b) Is there any pollen immigration into the area? (c) What pattern of pollen dispersal can be observed? and (d) How many seed trees are required for seed collection to supply ex situ reforestation, breeding, and forest restoration?

## 2 MATERIALS AND METHODS

### 2.1 Study site

**Figure 1:**
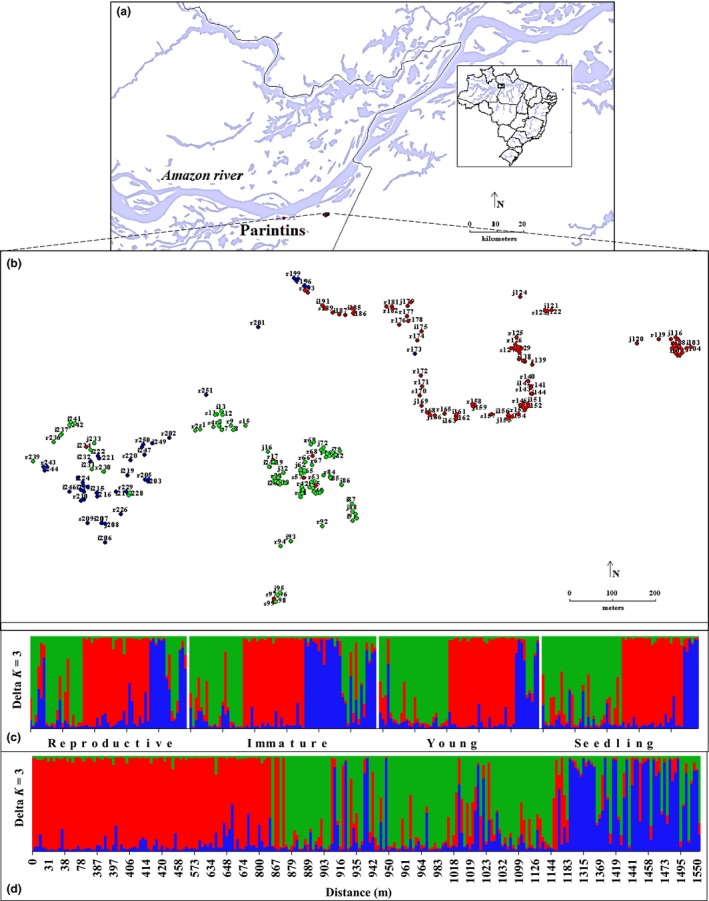
Sampling area of *Euterpe precatoria* in the Amazon forest, Brazil, and the Bayesian analysis. (a) Sampling area of the natural population used in this study. (b) Distribution of different plants sampled of each ontogenetic stage (r = reproductive; i = immature, J = young, and s = seedling) with ID number in situ (from 1 to 248) and colors indicating the result obtained in the structure. (c) Graph result of *K *=* *3 obtained in the analysis of the genetic structure of the 248 sampled plants, separated by ontogenical stages. (d) Chart result of *K *=* *3 of the 248 plants sampled and grouped based on the distances. Map constructed with DIVA‐GIS, version 7.5 (Diva‐Gis, 2012)

This study was conducted in a natural population of *E. precatoria*, growing in a rural area called *Nossa Senhora de Fátima do Açaí*, of the rural community of *Vila Amazonia*, in the municipality of *Parintins*, Amazonas, Brazil (Figure 1a). At the geographic location of this population (latitude 02º 36′ 52.09″ S and longitude ‐56º 33′ 29.13″ W), the climate type is tropical monsoon—Am (Peel, Finlayson, & McMahon, 2007). The population is delimited at the four cardinal points (north, south, east, and west) by natural forest vegetation in between deforested mosaic fragments with different sizes, forming a landscape with large areas of native vegetation interrupted by deforested (cleared) areas. However, it is noteworthy that the Amazon River basin lies in the north, immediately behind the forest. This information about the sampling area shows the degree of distribution of the species in the native and preserved forests. The trees in the area are used by locals for fruit collection, since açaí is part of the diet of the population.

### 2.2 Sampling

In a 10 ha area, 248 plants were randomly sampled. The geographic coordinates of each sampled plant were recorded with a global positioning system equipment (GPS map 60CS—GARMIN). More than 70% of the reproductive trees of the species in the area were sampled and classified into four ontogenetic stages: (a) seedlings—plants with transitional leaves, between bifid and pinnate without stem, with heights of 0.30–1 m; (b) young plants—apparent stem (without leaf sheath), pinnate leaves, with heights of 1.01–2 m; (c) immature plants—with stems, but no sign of reproductive structures and heights generally from 2.01 to 4 m; (d) reproductive plants—with signs of previous reproductive events, with inflorescence or infructescence, and mostly higher than 4 m. The four ontogenetic stages and their respective plant heights were determined based on studies with *Euterpe edulis* (Portela & Santos, 2011) and *E. precatoria* (Rocha & Viana, 2004) and based on the concept of two phases of minimum light requirement for developing plants and adult plants (Kahn, 1986). In 2014, 59 seedlings, 60 young, 70 immature, and 59 reproductive plants were sampled. From each plant, one new leaflet was collected, which was individually stored in a previously labeled ziplock plastic bag, containing silica gel until the samples could be stored at −20°C in the Laboratory of Genetics and Plant Breeding of the Department of Agronomy of the *Universidade Federal do Amazonas* (UFAM).

### 2.3 Amplification of microsatellite markers

Total DNA was extracted with the cationic detergent CTAB (2% cationic hexadecyl trimethyl ammonium bromide), as proposed by Doyle and Doyle (1990) and then quantified in GelRed (Biotium) (Ramos et al., 2011). Eighteen microsatellite loci developed for *E. precatoria* (Epr01, Epr02, Epr04, Epr05, Epr06, Epr13, Epr14, Epr15, Epr18, Epr19, Epr21, Epr22, Epr27, Epr31, Epr32, Epr33, Epr35, and Epr36) were used in the study (Ramos, Dequigiovanni, Lopes et al., 2016). These microsatellites were amplified by polymerase chain reaction (PCR) in a Veriti Thermal Cycler (Applied Biosystems), in a total reaction volume of 10 μl, containing 10 ng genomic DNA, 1× buffer (10 ×  standard *Taq* reaction buffer), 210 μM of each dNTP, 1.5 mM MgCl_2_, 0.16 μM forward primers, and M13‐labeled primers (FAM, HEX, or NED dyes) (Schuelke, 2000), 0.32 μM reverse primers, 1.05 U *Taq* DNA polymerase (Invitrogen), and 3.49 μl ultra‐pure water. The PCR amplifications occurred in two phases, of which the first was primer‐specific and the second to connect the M13. The first stage began by stabilizing the temperature at 68°C for 2 min and at 92°C for 30 s, followed by 30 cycles (30 s at 92°C for denaturating, 35 s at the primer‐specific annealing temperature, and 35 s for extension; the second step consisted of 15 cycles (30 s at 92°C, 30 s at 53°C, 30 s at 72°C) and a final extension at 72°C for 15 min followed by a period of 15 min at 68°C (Ramos, Dequigiovanni, Lopes et al., 2016). Amplification products were checked by electrophoresis on 1.5 % agarose gels stained with GelRed (Biotium) in 1× TBE buffer (pH 8.0). Amplified PCR products were subjected to automated DNA analysis by capillary electrophoresis in an ABI 3130XL Genetic Analyzer (Applied Biosystems). The size standard GeneScan^™^ 500 ROX^®^ (Life Technologies) was used to determine the size of the alleles. Amplified fragments were observed and analyzed with software GENEMAPPER v4.0 (Applied Biosystems).

### 2.4 Analysis of genetic diversity and inbreeding

The genetic diversity of the ontogenetic stages of seedling, young, immature, and reproductive plants was compared estimating the following indices: total number of alleles per locus (*k*), mean number of alleles per locus (*A*), overall allelic richness (*A*_*r*_), average observed (*H*_*o*_), and expected (*H*_*E*_) heterozygosities. These indices were estimated using the *divBasic function* of package *diveRsity* (Keenan, McGinnity, Cross, Crozier, & Prodohl, 2013) of project R (R Core Team, 2016). The number of private alleles (*A*_*p*_) in each ontogenetic stage was estimated using the GDA program (Lewis & Zaykin, 2002). Inbreeding was estimated using the fixation index (*F*), and 1,000 Monte Carlo permutations of alleles among plants were used to test whether the *F* values were statistically different from zero, associated to a *Bonferroni* correction (95%, α = 0.05), using SPAGeDI 1.3 (Hardy & Vekemans, 2002). The Scott‐Knott test was used to detect significant differences between the mean values of *A*,* H*_*o*_, *H*_*E*_, and *F* for the four ontogenetic stages, after evaluating significant differences by univariate analysis in a completely randomized design (CRD). Previously, the homogeneity of variances of ontogenetic stages and data normality was tested by the Bartlett and Shapiro–Wilk tests, respectively. These analyses were performed using the functions *Bartlett.test*,* shapiro.test,* and *AOV* (CRD) of the R package and *LScottKnott* function of the package *laercio* (Silva, 2015) in project R (R Core Team, 2016).

Among the individuals sampled from each ontogenetic stage, a genetic structure analysis was performed due to the large area sampled. A Bayesian analysis was performed to determine the possible number of clusters of the set of sampled accessions (four ontogenetic stages) within a same area of research. Software Structure (Pritchard, Stephens, & Donnelly, 2000) was used based on the Admixture model, which is commonly applied to real or natural populations. The number of clusters (*K*) was set between 1 and 8, and for each *K*, 10 iterations were performed with a burn‐in of 100,000 followed by 200,000 MCMC iterations. The number of clusters was estimated based on the Evanno ΔK method, indicating that the most likely *K* is where the change is greatest in the second‐order rate of change in Pr(X|K) between successive *K* values (Evanno, Regnaut, & Goudet, 2005). Based on the selected *K* value, consensus of the iterations obtained in this cluster was made using the program CLUMPP v.1.1.2—Cluster Matching and Permutation Program (Jakobsson & Rosenberg, 2007) and the Distruct program. v.1.1 (Rosenberg, 2004), to graphically visualize the structure of each ontogenetic stage.

### 2.5 Analysis of SGS

The SGS was investigated independently for each ontogenetic stage (seedling, young, immature, and reproductive), using the estimate of the mean coancestry coefficient (*θ*_*ij*_) between pairs of plants in each ontogenetic stage, calculated as described by Loiselle, Sork, Nason, and Graham (1995) with program SPAGeDI 1.3 (Hardy & Vekemans, 2002). To visualize SGS of the four ontogenetic stages, the *θ*_*xy*_ values were plotted against 10 distance classes. Plants were defined by the program itself, from the number of plants sampled in each ontogenic class (Loiselle et al., 1995). To detect the significance of deviation of SGS from a random structure, the 95% confidence interval (95% CI) was calculated for each observed *θ*_*ij*_ value and each distance class, using 10,000 Monte Carlo permutations of plants from different distance classes. To compare the SGS between ontogenetic stages and other studies, the following statistic was used: *Sp* = −*b*_*k*_/(1 − θ_1_), where *θ*_*1*_ is the mean pairwise coancestry coefficient calculated among plants within the first distance class, and *b*_*k*_ is the slope of the regression curve of the mean coancestry coefficient in function of the logarithm of the spatial distance (Vekemans & Hardy, 2004). To test the intensive SGS, the spatial position of the plants was permutated 10,000 times to obtain the distribution frequency of *b*_*k*_. The null hypothesis indicated that *θ*_1_ and ln (*d*_*xy*_) are not correlated (*d*_*xy*_ is the spatial distance between plants *x* and *y*). These analyses were run on SPAGeDI 1.3.

### 2.6 Reproductive ontogenetic stage: coancestry and effective group size

The coancestry group (Θ) was estimated for reproductive plants by the method of Lindgren and Mullin (1998). The pairwise coancestry coefficient between all pairs of plants (*θ*_*ij*_) was calculated with the estimator described by Loiselle et al. (1995) and implemented in SPAGeDI: $\Theta =\left[0.5n\left(1+F\right)+\sum_{i=1}^{n}\sum_{j\neq i}^{n}\theta_{\mathrm{ij}}\right]n^{2}$, where *n* is the number of sampled plants and *F* the inbreeding coefficient of the population, estimated from the fixation index. The effective population size (*N*_*e*_) was calculated as proposed by Cockerham (1969), from the variation of gene frequencies due to genetic drift: *N*_*e*_ = 0.5/Θ. When estimating Θ and *N*_*e*_, negative *F* values were assumed as zero.

### 2.7 Analysis of pollen and seed dispersal patterns

For these analyses, the 59 plants of the reproductive ontogenetic stage were considered candidate parents. The other three ontogenetic stages were clustered and named regenerated plants (189 plants). Paternity analysis was based on the categorical maximum‐likelihood method, using CERVUS 3.0.3 (Kalinowski, Taper, & Marshall, 2007). The paternity of the regenerated *E. precatoria* plants was estimated by statistic Δ, calculated by simulations, considering 10,000 replications (simulated for the progenies), considering a maximum of three mismatches per locus and all 59 candidate parent palm trees as pollen donors for the regenerated plants (it was assumed that 50% of the pollen donors in the study area were sampled). We adopted a confidence level of 80% as suggested by Marshall, Slate, Kruuk, and Pemberton (1998) for the assigned paternity. Self‐fertilization was also considered possible and was estimated. The pollen immigration rate (*m*_*p*_) into the area was estimated as the number of progenies for which no father was assigned in the sampled area, divided by the total number of sampled regenerated plants. The pollen dispersal distance was calculated between all reproductive ontogenetic stage palm trees as possible candidate parents and putative pollen donors. The distance of seed dispersal among the progenies was calculated as well as the closest distance to one of two possible candidates for putative parents. These distances of pollen and seed dispersal were calculated from the Euclidean distance between two points. The seed dispersal distance was also calculated independently for each one of the progenies (seedling, young, and immature ontogenetic stages).

To find whether the reproduction patterns were related to the distance between plants, the frequency of pollen dispersal curve was compared with the spatial distance among all possible parents by the Kolmogorov–Smirnov test (Sokal & Rohlf, 1995). The effective neighboring pollination area (*A*_*ep*_) was calculated assuming a circular area around a central seed tree (candidate parent), by $A_{\mathrm{ep}}=2\pi \sigma_{p}^{2}$ (Levin, 1988), where $\sigma_{p}^{2}$ is the axial variance of pollen dispersal.

The probability of exclusion of pairs of parents, *P*_*p*_ (Dow & Ashley, 1996), was estimated with program NM+ (Chybicki & Burczyk, 2010). Pollen flow, selfing, and pollen dispersal distance were also estimated assuming an exponential power dispersal Kernel (Austerlitz et al., 2004) with program NM+ (Chybicki & Burczyk, 2010), based on the neighborhood model (Burczyk, Adams, Moran, & Griffin, 2002). In this model, the distances of pollen dispersal and patterns were not derived from individual paternity assignments, as in the case of CERVUS, but indirectly from a spatially explicit mating model (Ramos, Dequigiovanni, Sebbenn et al., 2016). The model defines that the paternity of a progeny may result from: (a) self‐fertilization with probability *s*; (b) pollen immigration from outside the plot, with probability *m*_*p*_; or (c) crossing with a male located within the plot, with probability 1‐*s‐m*_*p*_ (Burczyk et al., 2002). The NM + was combined with the initial settings using categorical paternity assignment for our study plot. The neighborhood parameter was set to “infinity,” considering all sampled parent candidates of the study area as the neighborhood size (Chybicki & Burczyk, 2010). Pollen dispersal was modeled using the exponential power family parameter (Austerlitz et al., 2004; Chybicki & Burczyk, 2010) with estimates given by scale (*a*) and shape (*b*) of the parameters by which the mean distance of pollen and seed dispersal (δ) were estimated.

## 3 RESULTS

### 3.1 Genetic diversity

The 18 microsatellite loci used provided a number of alleles per locus varying from 4 (Epr22) to 18 alleles (Epr06 and Epr19), with a mean of 10.7 alleles. A total of 193 alleles were identified, distributed in 248 plants composing the four ontogenetic stages (reproductive, immature, young, and seedling). The number of alleles per locus among the 248 plants varied from 3 to 19, with a mean of 8.1. In the reproductive plants’ ontogenetic stage, the alleles per locus varied from 3 to 11, with a total of 125 and mean of 6.94 (Table 1). For the immature class, alleles per locus varied from 3 to 15, with a total of 135 and mean of 7.50. The young and seedling stages had a total of 145 and mean of 8.06 alleles, and from 3 to 16 and 3 to 15 alleles per locus, respectively. *A*_*r*_ presented values of 6.02 (3.00–9.13), 6.32 (2.58–12.00), 7,10 (3.00–14.67), and 6.88 (3.00–11.88) for reproductive, immature, young, and seedling ontogenetic stages, respectively (Table 1). All ontogenetic stages showed private alleles (Table 1), suggesting pollen immigration from other more distant areas. The observed heterozygosity (*H*_*o*_) was slightly lower than the expected heterozygosity (*H*_*E*_) for the reproductive plants and seedlings, and similar for the immature and young ontogenetic stages. The mean fixation index (*F*) was negative (reproductive and young plants stages) and very close to zero when positive (immature plants and seedling), indicating the absence of inbreeding. Compared individually, the genetic diversity indices of *A*,* H*_*o*_, *H*_*E*_, and *F* were not significantly different in the four ontogenetic stages.

**Table 1:** Genetic diversity indices for ontogenetic stages of *Euterpe precatoria*

Stage	*n*	*k*	*A*_*r*_	*A*_*p*_	*A*	*H*_*o*_	*H*_*E*_	*F*	*P*_*p*_
Reproductive	56	125	6.02	7	6.94	0.57	0.58	−0.021	0.99999994
Immature	67	135	6.32	7	7.50	0.58	0.58	0.019	
Young	57	145	7.10	15	8.06	0.62	0.62	−0.002	
Seedling	56	145	6.88	14	8.06	0.59	0.61	0.044	

*Note*

*n*—sample size; *k*—total number of alleles per locus; *A*_*r*_—overall allelic richness; A_p_—number of private alleles in each ontogenetic stages; *A*—mean number of alleles per locus; *H*_*O*_ and *H*_*E*_—observed and expected heterozygosity, respectively; *F*—fixation index; *P*_*p*_—probability of exclusion of pairs of parents.

When genetic analyses were performed for each ontogenic stage, the estimated number of genetically homogenous populations (*K*) in the Bayesian analysis determined *K *=* *3, indicating the existence of three populations within this area. In each ontogenetic stage, these three groups were constant (Figure 1b–d).

### 3.2 Spatial genetic structure

**Figure 2:**
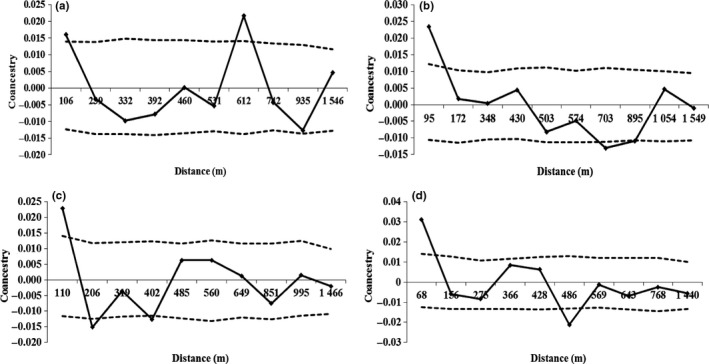
Correlogram of the coancestry coefficient (*θ*_*ij*_) for reproductive (a), immature (b), young (c), and seedling stages (d). The continuous line represents the mean *θ*_*ij*_ of value pairs, and the dashed lines the 95% confidence interval

The SGS was significant in the first distance class for the four ontogenetic stages (Figure 2, Table 2), suggesting that plants within this distance are relatives, because this distance was delimited within the expected for first cousins (*θ*_*ij*_ **= 0.0625). In addition, the coancestry levels were very similar, ranging between 95 and 110 m (for the reproductive, immature, and young stages) and 68 m for the seedling stage. The statistic that measures the extension of the SGS in the first distance class (*Sp*) for the ontogenetic stages showed a low intensity of SGS [*Sp* = 0.0053 (reproductive), 0.0085 (immature), 0.0068 (young), and 0.0089 (seedling)]. A reduction to negative *θ*_*ij*_ values was observed at a distance close to 239 m (reproductive), 156 m (seedling), and 206 m (young), while in the immature ontogenetic stage this distance was greater than 430 m. The *θ*_*ij*_ level was significantly lower than the lower limit of CI of 95% at short distances for the ontogenetic stage young (Figure 2). Above this distance, the *θ*_*ij*_ values were not significantly different from zero or substantially less than zero. The slope of the *b*_*k*_ regression of the pairwise coancestry coefficient in relation to the logarithm of the spatial distance scale for each ontogenetic stage was significantly negative for *b*_*k*_ (Table 2), showing a pattern of seed dispersal according to the isolation by distance. The *Sp* statistics were calculated for each ontogenetic stage (Table 2).

**Table 2:** Parameter estimates of the intrapopulation spatial genetic structure and effective size of *Euterpe precatoria* in four ontogenetic stages

Stage	*θ*_*1*_	fdc	*θ*_*1*_ 95% CI	*b*_*k*_	*S*_*p*_	Sds (m)	*n (N*_*e*_ *)*
Reproductive	0.0161[Link]	106	−0.0123 to 0.0139	−0.00518[Link]	0.0053	0–1,546	59 (20)
Immature	0.0236[Link]	95	−0.0106 to 0.0122	−0.00832[Link]	0.0085	0–1,549	70 (23)
Young	0.0228[Link]	110	−0.0115 to 0.0140	−0.00664[Link]	0.0068	0–1,466	60 (23)
Seedling	0.0312[Link]	68	−0.0145 to 0.0140	−0.00858[Link]	0.0089	0–1,440	59 (20)

*Notes*

*θ*_*1*_—coancestry coefficient in the first distance class; fdc—first distance class; CI—confidence interval; *b*_*k*_—slope of the regression curve of the logarithm of the spatial distance of the mean coancestry coefficient; *Sp*—statistic that measures the extent of the spatial genetic structure in the first distance class; Sds—spatial distance scale; *n*—number of plants in each ontogenetic stage; *N*_*e*_—effective size.

*p* < 0.05.

### 3.3 Group coancestry and effective population size

The group coancestry coefficients (Θ) for the four ontogenetic stages were as follows: reproductive (Θ = 0.025), immature (Θ = 0.021), young (Θ = 0.022), and seedling (Θ = 0.023). The effective size indicates that the 59 reproductive plants correspond to 20 (*N*_*e*_) unrelated and non‐inbred individuals (*N*_*e*_ */n *=* *0.34). For the other three ontogenetic stages, the results were similar to the first (Table 2).

### 3.4 Pollen and seed dispersal patterns

In the paternity analysis, the probability of exclusion of pairs of parents (Table 1) was high (*P*_*p*_ **= 0.99999994), indicating that pollen dispersal distances are partial, due to the probability that the true pollen donor is located within the population in the assessed continuous forest.

Of the 189 regenerated plants sampled, 107 (56.6%) were identified as originating from two parents from within the sampled population. Eight progenies were generated by selfing (*s* = 4.2%). The remaining 74 progenies were probably derived from pollen donors from outside the sampling area, suggesting a pollen immigration rate (*m*_*p*_) of 39.2%. The 107 outcrossing and eight selfing regenerated plants were apparently derived from 83.1% of the candidate parents from the reproductive ontogenetic stage plants.

**Figure 3:**
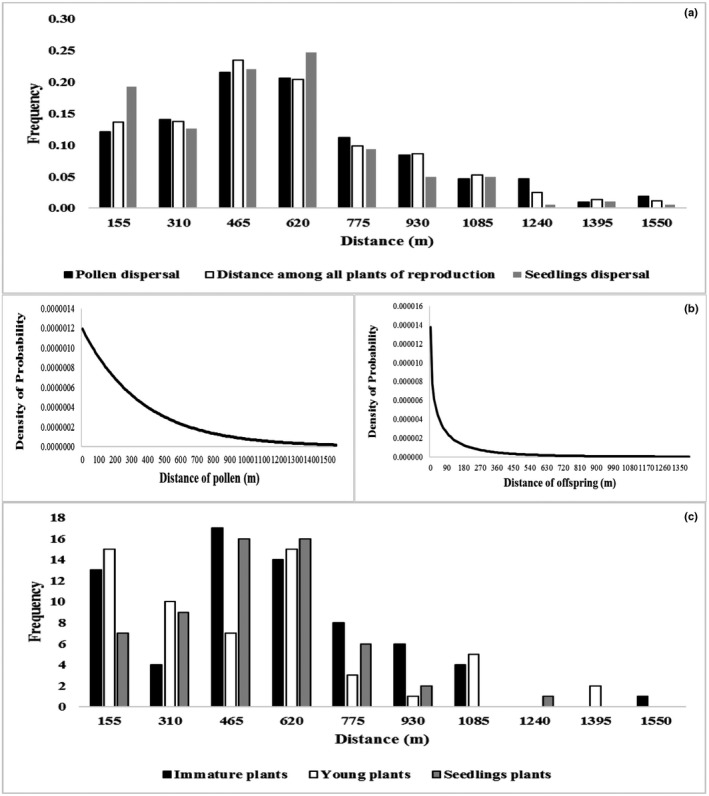
Pollen and seed (offspring) dispersal. (a) Effective frequency of pollen dispersal distance, the distance among all reproduction stage plants, and the seedling dispersal in the studied *Euterpe precatoria* population. (b) Estimated pollen and seed dispersal kernel. Scale and shape parameters estimated by the neighborhood model (Burczyk et al., 2002). (c) Frequency of pollen donors and seeds per distance

The pollen dispersal distance (δp) ranged from 46.4 to 1543.9 m, with a mean of 531.1 m (standard deviation of 316 m) and a median of 471.4 m (Figure 3a). The estimate of the correlation coefficient among the number of progenies fertilized by pollen of male progenitors (possible parents of the plants genotyped in this study) and the distance between mother plants within our sampling area was high and significantly different from zero ($R_{p}^{2}$ = 0.65, *p *<* *0.05), suggesting that the distance between plants had an influence on the mating likelihood.

When assessing the seed (189 progenies) dispersal observed in the study area, we identified 182 regenerated plants (96.3%) with at least one parent. The other seven regenerated plants were probably originated from outside our sampling area, suggesting a seed immigration of 3.7%. The seed dispersal distance (δs) ranged from 3.7 to 1,400.8 m, with a mean of 443.7 m (standard deviation 289.1 m) and a median of 428.4 m (Figure 3a). The correlation coefficient among the number of progenies and the distance closest to one of two possible candidates (possible mother plants) within our sampling area was high and significantly different from zero ($R_{s}^{2}$ = 0.70, *p *<* *0.05), suggesting that the distance between the possible parents influenced the probability distribution of the regenerated plants.

The Kolmogorov–Smirnov test was not significant (*D* = 0.067, *p* = 0.82; Figure 3a), indicating that the spatial distance between the trees explains the observed pollen dispersal pattern. The mean effective pollination neighborhood area was 62.74 ha, with a mean effective pollination radius of pollen dispersal in the vicinity of 446.91 m.

The dispersal kernel was fat‐tailed for pollen and slightly fat‐tailed for seeds (regenerated plants) (Figure 3b,c), indicating a high probability of long‐distance pollen dispersal and a somewhat limited and non‐random dispersal kernel of progenies. The NM+ program also found absence of selfing (*s* = 0), a rate of 0.4752 of pollen immigration and of 0.0057 of regenerated plants, a mean distance of 320.94 for pollen dispersal and 1,870 m for progenies, and a dispersal kernel scale of a = 365.59 (pollen) and *a* = 31.08 (progenies). The fat‐tailed shape was *b* = 1.0 (pollen), and the slightly fat‐tailed shape was *b* = 0.5 (progenies).

## 4 DISCUSSION

### 4.1 Genetic diversity

This is the first study in *E. precatoria* with specific microsatellite loci, addressing a paternity analysis, gene flow, genetic diversity, and SGS in different ontogenetic development stages of a natural spontaneous population in a climax forest. The genetic diversity analysis (Table 1) shows that *E. precatoria* is a species that maintains similar patterns of genetic diversity over generations, among reproductive, immature, young, and seedling ontogenetic stages. Other studies comparing parents and progenies in the species *Astrocaryum aculeatum* (Ramos, Dequigiovanni, Sebbenn et al., 2016), *Swietenia humilis* (Rosas, Quesada, Lobo, & Sork, 2011), *Copaifera langsdorffii* (Sebbenn et al., 2011), and *Hymenaea courbaril* (Carneiro et al., 2011) reported similar results to those obtained with *E. precatoria*.

The nonsignificant differences between the genetic parameters of the four ontogenetic stages could be related to the type of mating process of *E. precatoria*. It is a monoecious plant with morphological separation of male and female flowers in the inflorescences, contributing to avoid inbreeding. The floral phenology is also varied, which leads to a lack of synchronization due to protandry (Küchmeister, Silberbauer‐Gottsberger, & Gottsberger, 1997). The male flower anthesis lasts approximately 17 days, followed by a 6‐day break without any open male flower. Then, the female flower anthesis begins, with duration of 3 days. The total anthesis duration of the entire inflorescence lasts on average 25.5 days (Küchmeister et al., 1997). This information also indicates that the private alleles observed in the four ontogenetic stages would be a contribution of pollen donor plants from outside the study area, suggesting immigration of pollen from other areas.

The overall allelic richness for reproductive (6.02), immature (6.32), young (7.10), and seedling (6.88) ontogenetic stages of *E. precatoria* was similar to the juvenile (7.04 and 8.42) and adult individuals (6.18 and 7.57) ontogenetic stages, respectively, in the species *E. edulis* (Novello et al., 2018). In all generations, *H*_*o*_ and *H*_*E*_ had similar values, indicating that the genetic diversity found in the evaluated generations of this natural population in a protected and preserved area can be conserved. These heterozygosity values also indicate a low presence of inbreeding, which could be confirmed by the results of the fixation index (*F)*. Both heterozygosity and *F* values showed the presence of possible selfing events (immature and seedling) in the sampled plants. Nevertheless, this information contradicts the concepts of floral morphology and protandry described above. However, overlapping flowering periods were observed in other palm tree species and geitonogamy seems to be relatively frequent (Küchmeister et al., 1997). These *H*_*O*_, *H*_*E*_, and *F* values were already reported in a previous study with *E. precatoria,* which addressed the genetic parameters of different ontogenetic stages based on two interspecific microsatellites (Kageyama et al., 2004).

The Bayesian analysis showed that three different clusters of *E. precatoria* populations (*K *=* *3) were identified in all sampled plants (four ontogenetic stages). Moreover, we observed that these three clusters were constant in all ontogenetic stages (Figure 1b–d). This result showed that the distribution of the ontogenetic stages (immature, young, and seedling) is always close to their potential genitors (reproductive ontogenetic stage) and indicates how the dispersion capacity of the plant material and the mating system of the species and its allelic diversity (Leducq et al., 2011) influenced the clusters observed in the different spaces. Thus, regardless of the ontogenetic development, the genetic structure of the species shows that the sampled plants were established in a given space, very near the seed tree (Figure 1d). It is noteworthy that *E. precatoria* is a species with morphological characteristics of reproduction which is typical of outcrossing plants (Küchmeister et al., 1997) and that the gene flow between plants classified as reproductive will influence the genetic structuring process. Another variable that may have influenced the observed genetic structure would be the types of dispersion in *E. precatoria*. When plants disperse to nearby or distant locations, the genetic distance will be a possible variable of dissimilarity in the composition of the population (Kristiansen et al., 2012). The primary seed dispersal pattern of *E. precatoria* observed in situ is by rain, usually concentrated under the canopy, within a radius of 4 m. Secondary dispersion occurs at harvest by extractive farmers, with transport over long distances. Transport over shorter distances is the result of the action of rats and other rodents, while dispersion over longer distances occurs by birds such as toucans (Ramphastidae), Jacus (Cracinae), Arapongas (Cotingidae), and Sabias (*Turdus* sp.) (Bovi & Castro, 1993; Helmut, 1997). The seeds of *E. precatoria* are also dispersed by the river waters (floods), mainly along river banks (Bovi & Castro, 1993). Another concept that should be evaluated in the progenies (immature, young, and seedling ontogenetic stages) of *E. precatoria* is the survival success, which also depends on the habitat class where the seed will be dispersed, since an inadequate habitat can limit gene migration and gene flow (Pannell & Fields, 2014). This is particularly true for tropical forests, which contain a variety of environments, for example, dense and open forests, flooded and not flooded forests (“dry land”) and a wide variety of plant formations and transition zones (Katz et al., 2012).

### 4.2 Spatial genetic structure

The SGS detected among the four ontogenetic stages of *E. precatoria* suggests a seed dispersal pattern of isolation by distance, because pollen and seed dispersal were primarily near the trees of the reproductive class. This fact is related to the behavior of the species in the process of primary seed dispersal by rain, and secondary dispersal mainly by rodents and birds, which deposit seeds at short or long distances from the plants (Bovi & Castro, 1993; Helmut, 1997). This behavior of SGS is similar to the result of the Bayesian analysis, because in both analyses the plants located spatially close are generally relatives (Hardy & Vekemans, 2002).

The coancestry levels (Figure 2) in the first distance class of *E. precatoria* may be related to the recruitment and distribution process of the *E. precatoria* population given by the species dispersal behavior in a climax forest. Another interpretation of the result of SGS would be that tropical forests form a mosaic of environmental conditions, with varying distribution and magnitude between space and time, leading to a rich environmental heterogeneity that allows the maintenance of a high level of species diversity, with regeneration and the generation of differences in organism performance at different environmental scales (Otárola & Avalos, 2014). Thus, the widely distributed species such as *E. precatoria* (Kageyama et al., 2004) are exposed to a wide range of environmental conditions that should express plasticity of functional and demographic characters which lead to local adaptation, and this behavior is recurrent in tropical palms, where the species distribution and population dynamics are explained by these adjustments to environmental changes (Otárola & Avalos, 2014).

Thus, the extent of trait expression within an *E. precatoria* population varies according to the temporal and spatial scales. However, the species is classified as a climax forest species in view of the characteristics slow growth, high moisture requirement, low light requirement for seedling growth, a low plant survival rate, and long seedling phase (Bovi & Castro, 1993). This can explain the shorter distance to the seedling stage of *E. precatoria*, because the germination process begins shortly after seed dropping, with 50%–60% germination under climax forest conditions (Otárola & Avalos, 2014).

The *Sp* had a low intensity of SGS, which suggests a higher pollen dispersal distance in low‐density populations (Vekemans & Hardy, 2004). These results were also similar to those found in populations with high plant density per square meter, as in the species *Dicorynia guianensis* (*Sp* = 0.026) and *American vouacapoua* (*Sp* = 0.012) (Hardy et al., 2006), indicating that the population density is a major determinant of the SGS.

### 4.3 Group coancestry and effective population size

The Θ suggests that the plants of the reproductive as well as of the immature, young, and seedling ontogenetic stages were generated by random crossing and the inbreeding level is expected to be low (<3%) (Sebbenn et al., 2011), that is, the expected inbreeding rate by mating among relatives is very low.

Due to the low Θ, the effective population size (*N*_*e*_ **= 20) was low (Silva et al., 2011), showing that *E. precatoria* has a high proportion of related plants within the entire sampled area of the population. This was confirmed by results of the Bayesian analysis (Figure 1) and SGS (Figure 2), which both show the degree of kinship in clusters and distances of the sampled plants. Also, the dispersal and propagation of this species would be related with the process of colonization of the population, with *E. precatoria* plants with different levels of kinship within this area. This behavior of *N*_*e*_ was similar in the other ontogenetic stages, as observed in the results.

The SGS of each ontogenetic class indicated a high proportion of kinship within the population, due to the seed dispersal behavior over short and long distances.

However, the pollen immigration rate (39.1%) is sufficient to always maintain the genetic diversity, effective size and counteract possible effects of the degree of kinship that may lead to negative effects such as genetic drift.

### 4.4 Gene flow and dispersal patterns

According to the sampled plants of *E. precatoria,* the pollen flow entering the population was high (39.1%). This result could be influenced by animals as the main vector of pollen dispersal, pollinating the different plant species in tropical forests (Degen & Sebbenn, 2014). This pollination strategy could also be influencing the results of Bayesian analysis and SGS, because wind‐pollinated species generally have lower genetic differences between populations than species pollinated by animals (Degen & Sebbenn, 2014). This pollen flow may also be a contribution to the preservation of the genetic diversity of the study population, mainly by the introduction of new alleles (Sebbenn et al., 2011). Research on gene flow in other natural populations of tree species reported high immigration rates, as, for example, in *Symphonia globulifera* with a pollen immigration rate of 49% (Carneiro, Degen, Kanashiro, Lacerda, & Sebbenn, 2009) and of 61.3% in *Theobroma cacao* (Silva et al., 2011).

The pollen (δp) and seed dispersal distances (δs) (1,543.9 and 1,400.8 m, respectively) showed a dispersal flux over long distances within the studied population. However, these observed patterns of dispersal distance could possibly indicate a pattern of isolation by distance (Figure 3), since the correlations ($R_{p}^{2}$ = 0.65; $R_{s}^{2}$ = 0.70) were moderate. This result would be related with the patterns of primary and secondary dispersion explained above, as well as with pollination, since *E. precatoria* flowers are visited by a large number of visitors. The most constant visitors of both flower types were of the beetle families Staphylinidae, Chrysomelidae (*Halticinae sp*.), and Curculionidae (*Ozopherus muricatus*,* Cholus* sp., and *Phyllotrox* sp.) and bees of the family Halictidae. Apart from these, other beetles (families Scarabaeidae, Cerambycidae, Elateridae, Brenthidae, Orthoperidae, and Dermestidae) and bee species (families Apidae and Anthophoridae) visited the flowers less frequently or in lower numbers than the above families (Küchmeister et al., 1997). These insects have the potential to transport pollen over long distances (Goodwillie, Kalisz, & Eckert, 2005). This would explain the high mean area of effective pollination observed in the neighborhood (62.74 ha), confirmed by the Kolmogorov–Smirnov test (Figure 3a). The seeds of tropical forest trees can be dispersed by animals and by the river waters (Bovi & Castro, 1993; Helmut, 1997; Leite, Brancalion, Guevara, & Galetti, 2012) as mentioned above, which would explain their distribution.

In addition, the mean distance (471.4 m; *SD* = 316 m) of pollen dispersal of pollinating insects shows that *E. precatoria* would be a species with low density in a climax forest. However, it is considered a high‐density species (Kageyama et al., 2004), with more than 5 trees/ha, the minimum for such species (Degen & Sebbenn, 2014). High‐density species are pollinated by insects within distances of <100 m, and the maximum pollen dispersal distance of high‐density populations rarely exceeds 300 m in forests (Degen & Sebbenn, 2014). However, considering that the gene flow (pollen and seed dispersal) of *E. precatoria* is the result of the action of different animals, the distance may be greater than expected for high‐density species. Nevertheless, the clusters obtained with the Bayesian analysis (*K *=* *3) and the distances of plants with kinship in SGS show that the behavior of *E. precatoria* would be according to the expected for high‐density populations, suggesting that this pollen dispersal behavior is probably a general pattern of animal‐pollinated plants (Degen & Sebbenn, 2014).

One could consider that the male and female flowers share the same inflorescence and *E. precatoria* has mandatory cross‐fertilization with protandry, resulting in no inbreeding due to the strong temporal separation of anthesis of male and female flowers on the same inflorescence and because there is never more than one per inflorescence in anthesis per plant. Nevertheless, selfing was detected among our samples. In other palm species with overlapping periods of inflorescence flowering, protandry seems to be relatively frequent. For example, *Geonoma ininterrupta* (Listabarth, 1993) have protandrous inflorescences.

The behavior of the exponential power distribution of intermate distances within our study plot for pollen flow was similar to a slightly leptokurtic shape (*b* = 1.0) and the behavior of offspring (seeds) to a leptokurtic shape (*b* = 0.5). This indicates some long‐distance mating events. Similarly, the dispersal kernel inferred for *E. precatoria* using the spatially explicit neighborhood model showed a distribution with high probability of dispersal at low distances. However, a faster decline in dispersal probability with increasing distance was observed for offspring than for pollen. This behavior in the offspring indicates that the distance has a significant effect on motherhood. However, the behavior observed in pollen indicates that distance has no significant effect on all successful male matings. Similar long‐distance dispersal events at pollination and overall movement of pollen and seeds have been reported in recent studies, for example, in *Astrocaryum aculeatum* (Ramos, Dequigiovanni, Sebbenn et al., 2016) and *Phoenix canariensis* (Saro, Robledo‐Arnuncio, González‐Pérez, & Sosa, 2014).

The results found for *E. precatoria* would indicate that the genetic data used for the underlying dispersal model, which typically assumes a long tail of dispersal (Ottewell, Grey, Castillo, & Karubian, 2012), are in agreement with the model. The tail shape of the dispersal kernel (i.e., whether thin‐ or fat‐tailed) affects the ultimate distribution of genetic diversity within and between populations. Some studies report that pollen dispersal kernels are fat‐tailed in tree species (Austerlitz et al., 2004; Klein, Lavigne, & Gouyon, 2006; Matter, Kettle, Ghazoul, Hahn, & Pluess, 2013; Ramos, Dequigiovanni, Sebbenn et al., 2016; Saro et al., 2014).

### 4.5 Implications for species conservation, cultivation, and seed harvest

**Figure 4:**
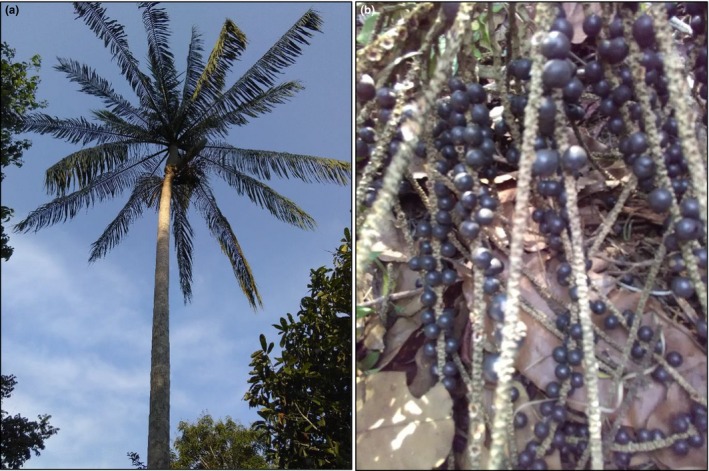
The palm (a) and bunches with fruits (b) of *Euterpe precatoria*

The results of this study have important implications for seed collection strategies (Figure 4b) for ex situ conservation, and for commercial reforestation with *E. precatoria* (Figure 4a). The presence of a SGS in the population indicates that seeds for ex situ conservation or for planting of production areas should be taken from reproductive plants spaced more than 110 m apart, to reduce the likelihood of occurrence of relatedness between the seeds harvested from different reproductive plants, and to avoid a reduction in the effective size of the sampled regenerated plants group. The inclusion of seeds from different unrelated reproductive plants in germplasm banks increases the effective size of the remaining population (Bittencourt & Sebbenn, 2007). However, the SGS shows that 83.05% of the pollen of the study population is dispersed to a distance of up to 471.4 m (average pollen dispersal distance). It can therefore be expected that the seeds represent a mixture of half‐ and full‐sibs, aside from having some level of inbreeding resulting from inbred crosses (Bittencourt & Sebbenn, 2007). Thus, the estimates of genetic parameters in progeny tests with seeds of this population must be corrected to take correlated and inbred crosses into consideration. Finally, pollen immigration from other populations is important because it can increase the genetic diversity and effective population size.

As conclusions of research, the sampled *E. precatoria* population has high levels of genetic diversity in the four evaluated ontogenetic stages (open‐pollinated reproduction, immature, young, and seedlings). The studied population has a large effective size, due to the SGS defined by seed dispersal over short and long distances of the plants of the reproductive stage, resulting in a high frequency of related plants within the population.

Pollen flow in the population occurs according to the isolation by distance model. Open pollination for seed formation can lead to some levels of inbreeding. Thus, the research on these genetic parameters of paternity analysis, pollen flow, and SGS for the different ontogenetic stages can be an effective way to assess the underlying impacts of harvest on the sampled population.

## DATA ACCESSIBILITY

The DNA sequences of the microsatellites, described by Ramos, Dequigiovanni, Lopes et al. (2016), are available: GenBank accessions KT198662‐KT198684. Individual sampling locations, morphological data (height), and microsatellite genotype data are available from the Dryad Digital Repository: http://datadryad.org/review?doi=doi:10.5061/dryad.7s7f7. The leaf samples used were stored in the Laboratory of Genetics and Plant Breeding of the agronomy department of *Universidade Federal do Amazonas*.

This research followed the norms of Resolution 21, of August 31, 2006—of CGEN (*Conselho de Gestão Do Patrimônio Genético*—*Ministério do Meio Ambiente*) (http://www.mma.gov.br/estruturas/sbf_dpg/_arquivos/res21cons.pdf). Material collection was registered in SISBIO (*Sistema de Autorização e Informação em Biodiversidade*/*Instituto Chico Mendes de Conservação da Biodiversidade*—*ICMBio*/*Ministério do Meio Ambiente*—*MMA*), voucher number 39950‐4.

## AUTHOR CONTRIBUTIONS

SLFR and PYK contributed to all stages, by planning the study design, sampling the populations, laboratory analyses, analyzing the data, and writing the manuscript. AMS contributed to the data analysis. MTGL, EAV, GD, AA, JLVM, and PPS contributed to the laboratory work. JLVM and PPS contributed to the sampling of the populations and contacts with farmers. All authors read and revised the manuscript.
